# Depending on Its Nano-Spacing, ALCAM Promotes Cell Attachment and Axon Growth

**DOI:** 10.1371/journal.pone.0040493

**Published:** 2012-12-10

**Authors:** Karsten Thelen, Steffen Jaehrling, Joachim P. Spatz, G. Elisabeth Pollerberg

**Affiliations:** 1 Department of Developmental Neurobiology, Centre for Organismal Studies, University of Heidelberg, Heidelberg, Germany; 2 Department of New Materials and Biosystems, Max-Planck-Institute for Intelligent Systems, Stuttgart, Germany; 3 Department of Biophysical Chemistry, University of Heidelberg, Heidelberg, Germany; US Naval Reseach Laboratory, United States of America

## Abstract

ALCAM is a member of the cell adhesion molecule (CAM) family which plays an important role during nervous system formation. We here show that the two neuron populations of developing dorsal root ganglia (DRG) display ALCAM transiently on centrally and peripherally projecting axons during the two phases of axon outgrowth. To analyze the impact of ALCAM on cell adhesion and axon growth, DRG single cells were cultured on ALCAM-coated coverslips or on nanopatterns where ALCAM is presented in physiological amino-carboxyl terminal orientation at highly defined distances (29, 54, 70, 86, and 137 nm) and where the interspaces are passivated to prevent unspecific protein deposition. Some axonal features (branching, lateral deviation) showed density dependence whereas others (number of axons per neuron, various axon growth parameters) turned out to be an all-or-nothing reaction. Time-lapse analyses revealed that ALCAM density has an impact on axon velocity and advance efficiency. The behavior of the sensory axon tip, the growth cone, partially depended on ALCAM density in a dose-response fashion (shape, dynamics, detachment) while other features did not (size, complexity). Whereas axon growth was equally promoted whether ALCAM was presented at high (29 nm) or low densities (86 nm), the attachment of non-neuronal cells depended on high ALCAM densities. The attachment of non-neuronal cells to the rather unspecific standard proteins presented by conventional implants designed to enhance axonal regeneration is a severe problem. Our findings point to ALCAM, presented as 86 nm pattern, for a promising candidate for the improvement of such implants since this pattern drives axon growth to its full extent while at the same time non-neuronal cell attachment is clearly reduced.

## Introduction

Cell adhesion molecules (CAMs) of the immunoglobulin superfamily (IgSF) are crucially involved in the development of the nervous system, e.g. by promoting axon growth and navigation [1]. Binding of IgSF-CAMs between contacting cell surfaces (trans-interactions) mediates cell attachment of various neuronal as well as non-neuronal cells [2]. Moreover, these interactions activate signaling pathways which regulate a multitude of cellular responses, including the behavior of axons and growth cones (i.e. the sensory tips of the axons) [3], [4]. In addition, IgSF-CAMs – most of them integral plasma membrane proteins - link the cytoskeleton to molecules in the environment and thus contribute to cell migration and axon advance [5]. It is widely assumed that for both processes, signaling and force generation, the density of IgSF-CAMs in the cell membrane and of their interaction partners in the environment of the cell is of pivotal importance [5]. Recently, a technique was developed which made it possible to analyze the impact of the spacing of protein domains on cellular functions by the use of regular arranged gold nanodot patterns with defined distances [6], [7]. Extracellular IgSF-CAM domains coupled to these nanodots in physiological density and orientation [8] as presented by cell surfaces can be used as cell culture substrates and the impact on cellular functions studied [9], [10]. Unspecific protein deposition in the interspaces between the nanodots is effectively prevented by passivation, thereby allowing for the undisturbed functional analysis of the nanodot-coupled protein domains.

The IgSF-CAM ALCAM (Activated Leukocyte CAM; previously also termed DM-GRASP, SC1, BEN, and JC7) is a highly conserved integral plasma membrane protein consisting of five extracellular Ig-domains, a trans-membrane domain, and a short cytoplasmic domain [11]. ALCAM interacts homophilically (i.e. with itself) and heterophilically (i.e. with IgSF-CAM L1/NgCAM and CD6) [12], [13], [14], [15], [16]. ALCAM has been shown to play a role in a variety of neuronal processes including cell adhesion [12], axon growth and navigation [16], [17], [18], [19] as well as migration [20], differentiation [21], and synapse formation [22], [23]. In the forming nervous system, ALCAM is selectively present on neurons carrying an axon and not found on neuroblasts or non-neuronal cells. ALCAM displays a spatially and temporally dynamic expression pattern; it is transiently enriched in far projecting, fast growing, and tract-forming axons during development of the visual system [18] and cerebellum [24]. Dorsal root ganglion (DRG) neurons also send out long axons which fasciculate and project into both the spinal cord as well as the body periphery; ALCAM is present on DRG axons and promotes their growth [9], [10], [17], [25]. Together, this points to the possibility that ALCAM might predominantly/specifically promote neuronal responses such as axon growth and not non-neuronal reactions (cell attachment e.g.).This would make ALCAM a candidate for the design of nerve conduits which mimick an axon bundle to support regeneration. ALCAM's potential impact on non-neuronal cells of the nervous system, however, had not been studied up to now.

In the present report, a coherent analysis of ALCAM's presence during DRG development is presented, revealing that the appearance of ALCAM on the two DRG axon populations correlates with their two growth phases. Using (among other substrates) nanopatterns, the impact of ALCAM's density on DRG neurons and non-neuronal cells was investigated: whereas ALCAM potently promotes axon growth also at low densities, it only moderately mediates non-neuronal cell attachment. Moreover, in-depth time-lapse analyses showed that the density of ALCAM affects growth cone morphology and dynamics as well as exploration behavior and advance efficiency.

## Results

### Expression of ALCAM during DRG development

**Figure 1:**
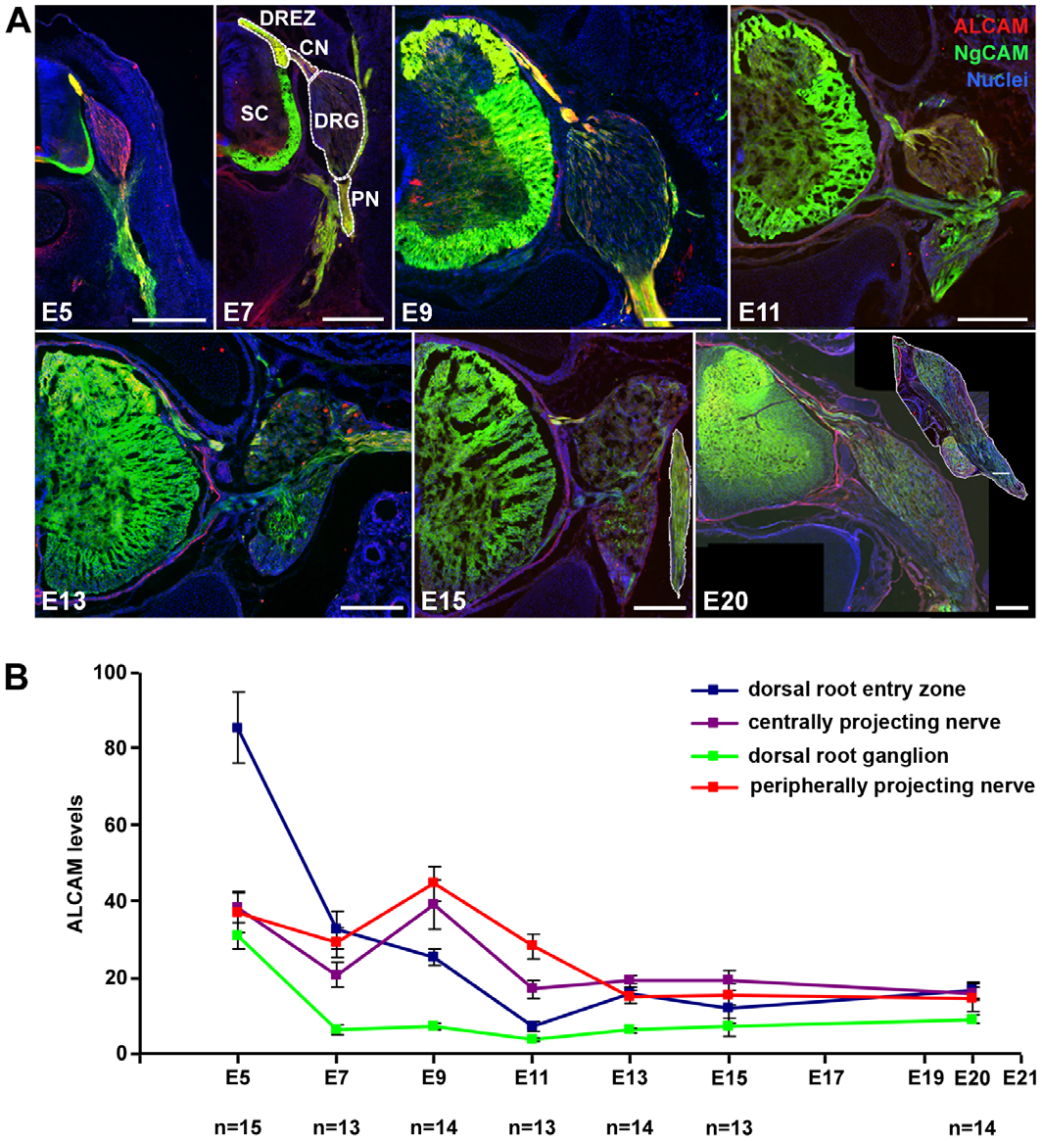
ALCAM distribution during DRG development. (**A**) Immunofluorescence labeling of ALCAM (NgCAM double staining for visualization of axons) in sections shows that -at E5- ALCAM is present in DRG, central nerve (CN), peripheral nerve (PN), and dorsal root entry zone (DREZ) and is absent from the spinal cord (SC) proper (except of ventral floor plate). At E7, ALCAM is predominantly present in the DREZ, and only at lower levels in PN and CN. At E9, levels of ALCAM are high again in the PN and CN and almost unchanged in the DREZ; within the DRG, ALCAM is only present in the dorso-medial part. At E11, E13, E15, and E20, all regions show only weak levels of ALCAM. Inserts in E15 and E20 show the PN. Scale bars: 250 µm. (**B**) Quantification of ALCAM levels by immunofluorescence intensity measurements (arbitrary units) in four DRG regions during embryonic development (E5–E20). Error bars represent SEM.

To analyze levels and distribution patterns of ALCAM protein in the developing spinal ganglia, thoracic DRGs were examined at various embryonic stages (**Fig. 1**). The dorsal root entry zone (DREZ) in the spinal cord as well as central nerve (CN; built up by axons projecting to the spinal cord) and peripheral nerve (PN; formed by axons projecting to the periphery) of the DRGs were also analyzed. ALCAM was labeled immunohistochemically in cross sections of embryonic day 5 (E5) to E20 (**Fig. 1a**) and fluorescence levels were quantified (**Fig. 1b**). DRG axons in DREZ, CN, and PN are strongly ALCAM-positive at E5, i.e. the time when the proprioceptive DRG neurons send out their axons into these structures. At this stage, ALCAM is also present in the DRG itself, in particular in the ventro-lateral region, i.e. the area where the somata and proximal axons of proprioceptive neurons locate, indicating ALCAM's presence in these neurons in this early phase of axon extension. At E7, the DRGs are almost devoid of ALCAM, pointing to a loss of this protein from the proximal axon region of proprioceptive neurons as well as the absence from the somata of all (other) neurons and non-neuronal cells (glial precursors, i.e. future Schwann cells and satellite cells). In contrast, the presence of ALCAM is maintained (at lower levels than at E5) in DREZ, PN, and CN, where massive axon fasciculation, i.e. DRG axons tracking on axons, is taking place at that stage. In DRGs, a second phase of axon extension (with the maximum at E9) takes place when the nociceptive neurons send out their axons. ALCAM is again strongly present in DREZ, PN, and CN. Within the DRG, only the dorso-medial part displays (low levels of) ALCAM; this is the region where the somata of the nociceptive neurons are situated, thus indicating the presence of ALCAM in the early phase also of the second wave of axon extension. From this developmental stage onwards, no further axons grow out from DRGs, and ALCAM quickly disappears from DRG, PN, and CN (E11–E20), i.e. from all axons and somata of all DRG cells. Together, this analysis revealed that in vivo the highest ALCAM densities are found on extending, bundling DRG axons and thus points to a role of ALCAM for axon tracking on pre-existing axons, hence in fasciculation.

### Impact of ALCAM density on DRG axon growth

**Figure 2:**
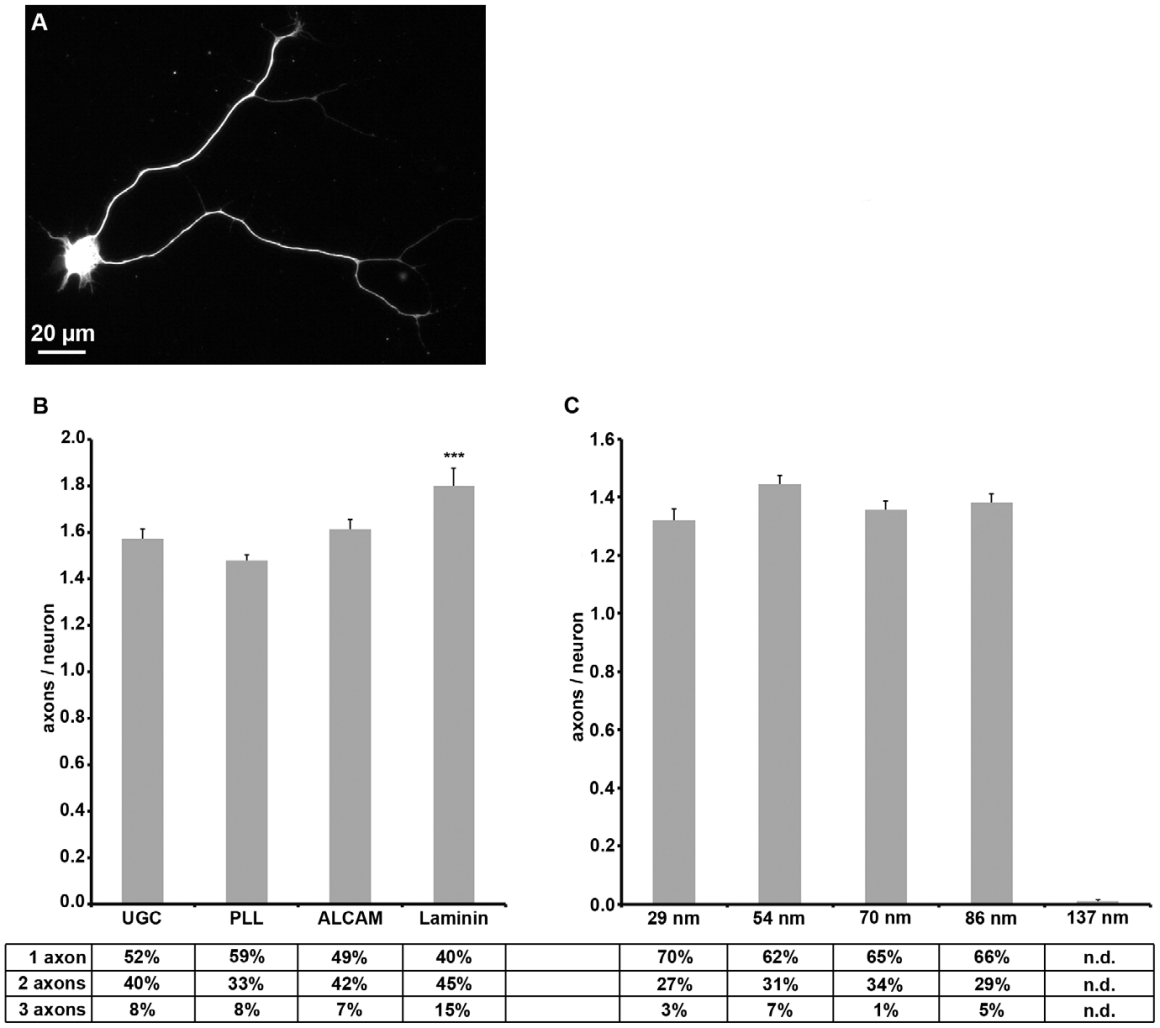
Axon formation on various substrates. (**A**) Immunofluorescence labeling shows a neuron (identified by β3 tubulin staining) with two axons. (**B**) Quantification of axons formed by a neuron on uncoated glass (UCG) or glass coated with various substrate molecules. Table indicates the proportion of neurons with one, two, or more axons (n.d. = not detected). (**C**) Quantification of axons formed by a neuron on various ALCAM nanopatterns. Error bars represent SEM; for statistical analyses, two-way ANOVA followed by post-hoc comparisons using two-tailed Student's t test with Bonferroni-Holm corrections were performed (***P<0.001, **P<0.01, *P<0.05).

We thus wished to gain insight into the role of ALCAM trans-interactions as take place between the contacting membranes of axons growing on axons [18], [19] for axon growth. For this, neurons in sparse E9 DRG single cell cultures were allowed to send out axons on ALCAM-coated coverslips (**Fig. 2**) and were triple-labeled after one day in vitro (1 div) for visualization of the axons. For comparison, the major extracellular matrix component laminin, the widely used substrate poly-L-lysine (PLL), and uncoated glass coverslips were also examined. On ALCAM-coated glass coverslips, about half of the DRG neurons sent out one axon, an almost equal fraction extended two axons, and only a few DRG neurons formed more than two axons, resulting in an average value of 1.6 axons/neuron (**Fig. 2a, b**). This reflects the neuronal morphogenesis in DRGs where at this stage the proprioceptive neurons possess already two axons whereas the nociceptive neurons are just forming their axon(s). A similar degree of axon formation was observed on PLL- and uncoated coverslips; this has to be attributed, however, to the axon promoting effects of proteins shed by DRG cells and deposited on the coverslip [26], [27], as also indicated by the axon formation on uncoated glass. This strong effect of shed proteins on axogenesis overrides the impact of ALCAM even if coated at a very high concentrations (up to 50 µg/ml, i.e. more than 100-fold density compared to physiological densities of ALCAM in the axonal plasma membrane [**9**]).

On ALCAM nanopatterns (Supplemental Information **Fig. S1**), in contrast, the deposition of shed proteins is prevented due to an effective passivation [6]; ALCAM is thus the only substrate molecule offered to the DRG cells (**Fig. 2c**). Moreover, ALCAM is spaced at a highly defined density and in the same orientation as presented by a cell surface (i.e. extracellular domain with the amino terminus up). DRG single cells were cultured on this substrate for 1 div and labeled as described above. The distance of ALCAM molecules varied from 29 nm (1,302 molecules/µm^2^) to 137 (62 molecules/µm^2^), the smallest distance representing a density of ALCAM which is in the range of the ALCAM density in the plasma membrane of DRG axons (about 1,600 molecules/µm^2^ [9]). The use of the nanopatterns revealed that ALCAM alone was capable of driving axon outgrowth if offered above a minimum density (137 nm spacing). The number of axons formed by a DRG neuron did not significantly differ on the various ALCAM nanopatterns: The majority of neurons formed one axon, about a quarter of the neuron population two axons, and the remaining minority more than two axons, resulting in average values ranging between 1.3 to 1.5 axons/neuron on the various nanopatterns. These data show that the number of axons formed by DRG neurons in vitro is largely independent of the type and density of the substrate, pointing to a strict intrinsic control of this parameter.

**Figure 3:**
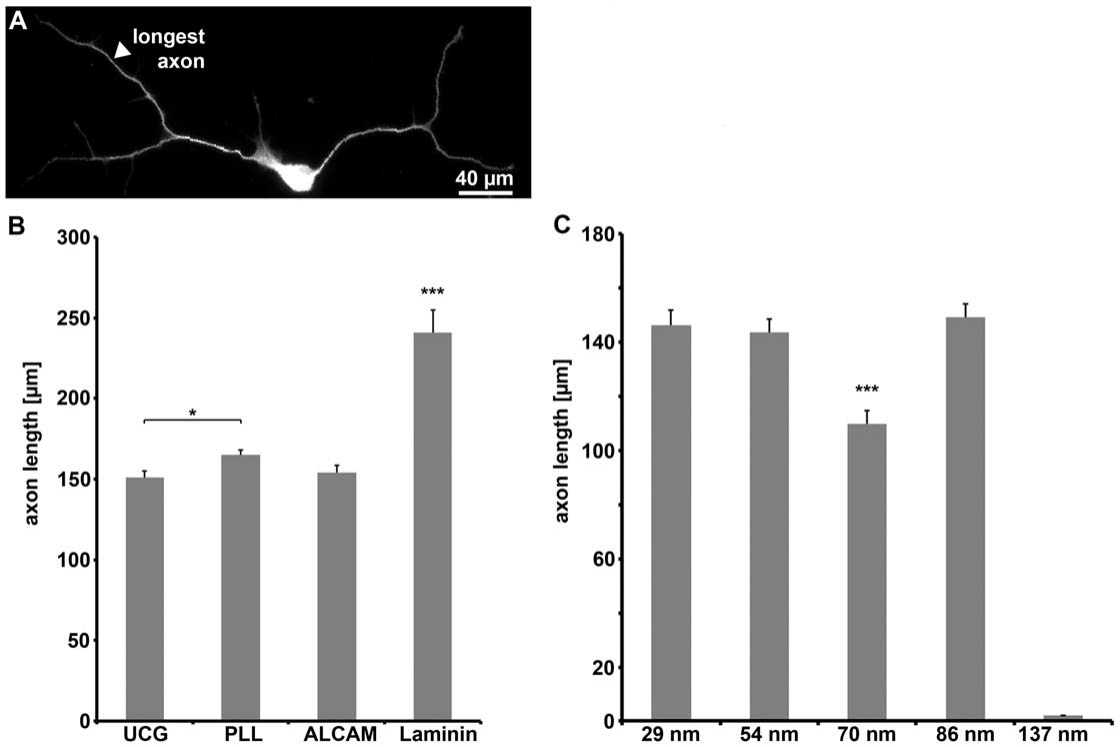
Axon length on various substrates. (**A**) Immunofluorescence labeling shows a neuron (identified by β3 tubulin staining) with two branched axons. (**B**) Quantification of length of the longest axon of a neuron (minimal length: 30 µm) on uncoated glass (UCG: n = 280) and glass coated with various substrate molecules (PLL: n = 833; ALCAM: n = 272; laminin: n = 104) or (**C**) various ALCAM nanopatterns (29 nm: n = 168; 54 nm: n = 463; 70 nm: n = 265; 86 nm: n = 402). As no differences in axon length on uncoated glass and glass coated with 50 µg/ml ALCAM were observed, axon lengths on glass coated with various ALCAM concentrations were not quantified.

To analyze the impact of ALCAM offered as a substrate on axon extension, the length of DRG axons in single cell cultures was determined (**Fig. 3**). Laminin, a known effective driver of DRG axon extension, was the strongest axon elongation-enhancing substrate (axon length: 241±14 µm, determined for the longest axon); on uncoated, PLL-, or ALCAM-coated coverslips, axon length values were lower (151±4 µm, 165±3 µm, and 154±1 µm, respectively) (**Fig. 3a, b**). The seeming lack of ALCAM impact on axon length (compared to PLL/uncoated glass) has to be attributed to protein deposition masking the impact of ALCAM as observed before (**Fig. 2**). ALCAM nanopatterns, in contrast, clearly revealed the capability of ALCAM to drive axon extension by itself, leading to axon lengths of about 140 µm (**Fig. 3c**). Above the threshold value (137 nm), the axon length was independent of substrate ALCAM densities which varied by a factor of almost ten (between 29 and 86 nm); a significant reduction of the axon length (110±5 µm), however, was found on nano-patterns with a spacing of 70 nm. This analysis revealed that ALCAM per se is a potent driver of axon growth, a prerequisite for the use of this CAM to enhance nerve regeneration (see Discussion).

**Figure 4:**
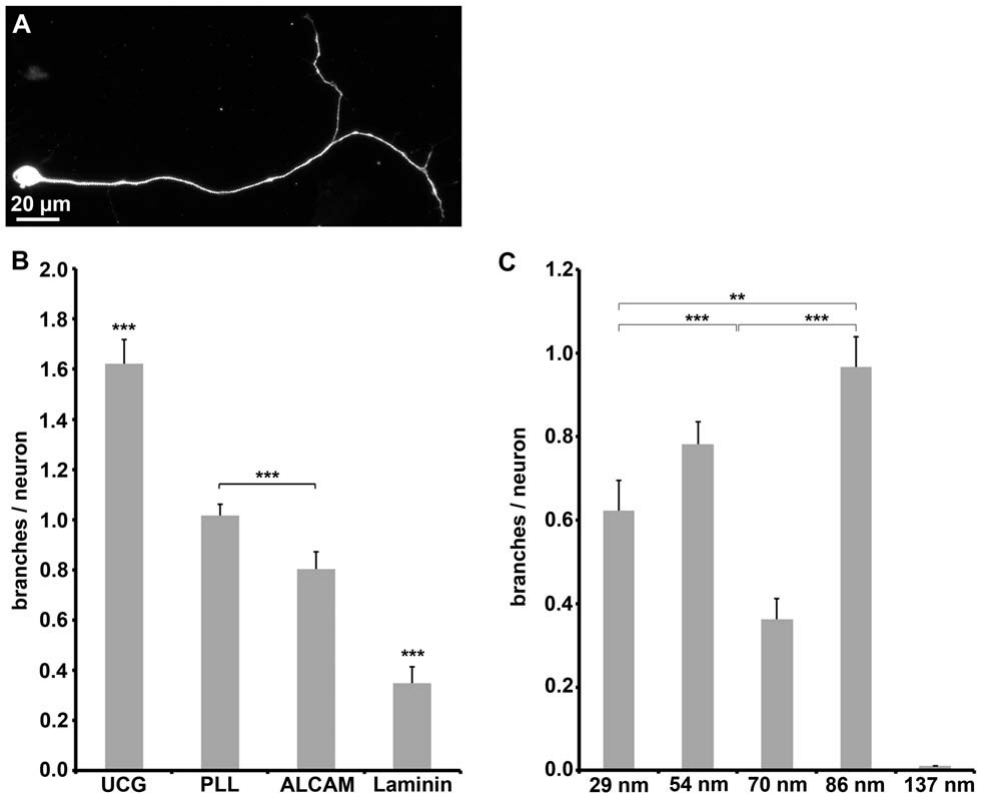
Axon branching on various substrates. (**A**) Immunofluorescence labeling shows a neuron (identified by β3 tubulin staining) with one branched axon. (**B**) Quantification of branches per neuron on glass coated with various substrate molecules. (**C**) Quantification of branches per neuron on various ALCAM nanopatterns. Error bars represent SEM; for statistical analyses, two-way ANOVA followed by post-hoc comparisons using two-tailed Student's t test with Bonferroni-Holm corrections were performed (***P<0.001, **P<0.01, *P<0.05).

The capability of ALCAM to govern axonal branching, which is also of relevance for regeneration paradigms, was also analyzed (**Fig. 4**). The number of branches emerging from the axons of DRG neurons (**Fig. 4a, b**) was highest on uncoated and PLL-coated coverslips (1.6±0.1 and 1.0±0.04 branches/neuron, respectively, and lower on ALCAM-coated ones (0.8±0.07). On laminin substrate, the number of branches was strongly reduced; branching was observed for only about every third neuron (0.35±0.07 branches/neuron) pointing to a potent branch formation suppressing property of laminin. Also ALCAM was capable of reducing branching, however in a more moderate fashion (minus 50% compared to uncoated glass and minus 20% compared to PLL-coated glass). ALCAM (coated on coverslips) inhibited axonal branching to a degree that makes the effect detectable despite of the impact of the shed proteins; in contrast, the effect of ALCAM (coated on coverslips) on axon formation or elongation was not detectable. ALCAM nanopatterns (**Fig. 4c**) revealed that the number of branches decreases with increasing ALCAM density (from about 0.6 to 0.9 branches/neuron on 29 nm and 86 nm patterns, respectively), demonstrating that ALCAM has a “dose-dependent” moderating impact on branch formation. On 70 nm patterns, fewer branches were formed (0.4 branches/neuron) than on the other patterns. Together, the data show that ALCAM per se is permits axon formation and elongation and at the same time suppresses branch formation.

### Impact of ALCAM density on growth cone behavior

**Figure 5:**
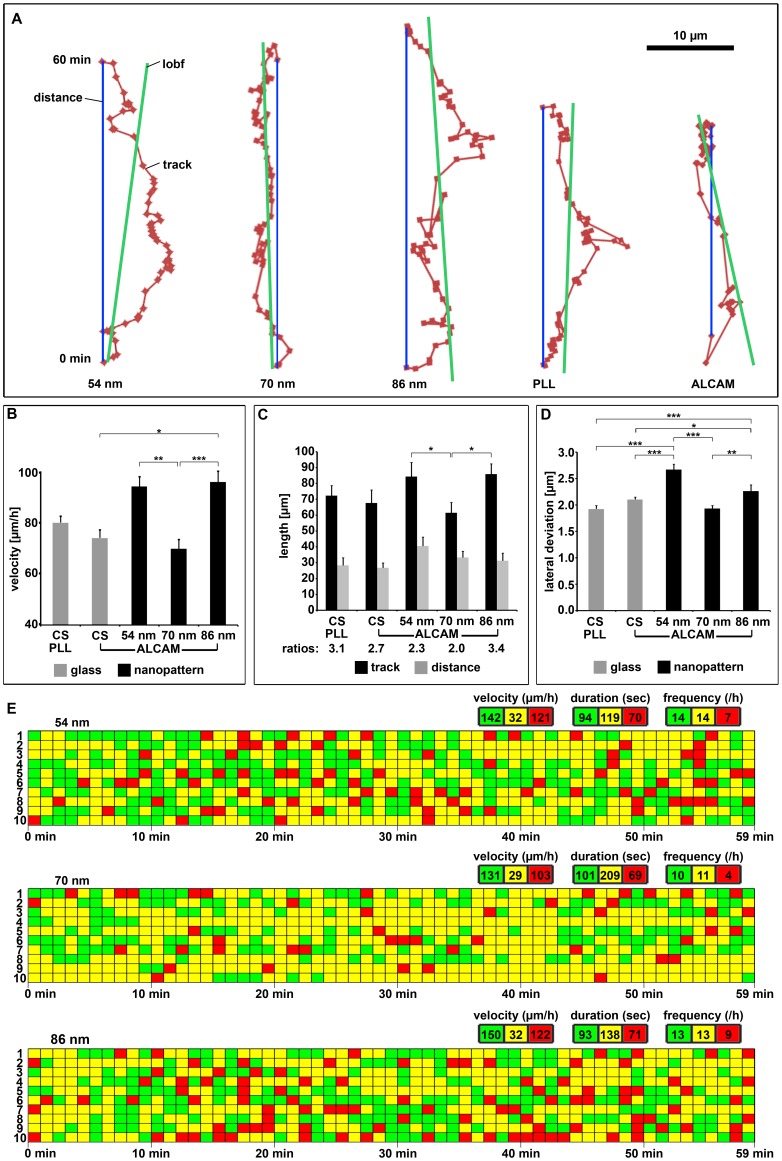
Growth cone behavior on various substrates. (**A**) Growth cone tracks (dark red) on ALCAM nanopatterns or PLL-/ALCAM-coated glass as observed by time-lapse phase contrast microscopy. Each dot represents the position of the growth cone neck (localized every minute); the blue line depicts the distance covered within the 60 min observation time and the dark green line the line of best fit (lobf, overall growth direction). For additional tracks, see Supplemental Figure S2. (**B**) Quantification of growth cone velocity (determined every minute as advance on line of best fit) on PLL-/ALCAM-coated glass or on ALCAM nanopatterns for ten growth cones each. (**C**) Quantification of track lengths and distances (see A) on various substrates for ten growth cones each. (**D**) Quantification of the lateral track deviations from the distance line (see A) per minute on various substrates for ten growth cones each. (**E**) Growth cone behavior, i.e. advance, pause, and retraction (green: >1 µm/min, yellow: −1 to +1 µm/min, and red: <−1 µm/min, respectively) on various ALCAM nanopatterns of ten different axons each, plotted for 60 min. Velocity, duration, and frequency of the three types of behavior were determined with respect to the line of best fit. For growth cone behavior on PLL/-ALCAM-coated glass, see Supplemental Figure S3. Error bars represent SEM; for statistical analyses, two-way ANOVA followed by post-hoc comparisons using two-tailed Student's t test with Bonferroni-Holm corrections were performed (***P<0.001, **P<0.01, *P<0.05).

To investigate the dynamics of axons and growth cones on the various substrates, time-lapse analyses of axons sent out by DRG neurons were performed (**Fig. 5**). For quantification of the growth cone's migration behavior, the position of the growth cone neck was determined every minute over a period of 1 hour and the resulting tracks plotted (**Fig. 5a**) (Supplemental Information **Fig. S2**; movies: please also see Supplemental Information **Movie S1, S2, S3, S4, S5**). On PLL- and ALCAM-coated coverslips, the velocity of the growth cones was about 75 µm/h (**Fig. 5b**); on ALCAM nanopatterns, growth cones were faster (almost 100 µm/h on 54 and 86 nm patterns) which is surprising since nanopatterns possess far less ALCAM (by a factor of 330 and 500 on 54 and 86 nm, respectively) than ALCAM-coated coverslips. The experiments reveal that growth cone advance benefits stronger from the highly defined, homogenous presentation of ALCAM (i.e. on nanopatterns) than axon branching which might be enhanced by local ALCAM aggregates (i.e. on coverslips) and is thus reduced on nanopatterns. Again, on 70 nm patterns, a lower performance than a linear correlation of growth cone velocity and ALCAM density would predict (see Discussion) was observed for growth cone velocity (70 µm/h).

To determine the efficiency of axon advance, we compared the length of the growth cone track to the distance line between start and end point within one hour (s. **Fig. 5a**). The growth cones track and the distance covered (i.e. also the advance efficiency) on the ALCAM nanopatterns were similar (**Fig. 5c**) except for the 70 nm pattern. This is due to a comparable degree of lateral wandering of the growth cone tracks from the distance line on 54 and 86 nm patterns (160 and 136 µm total lateral deviations/h, respectively) (**Fig. 5d**). On the 70 nm patterns, the lateral deviations are reduced (116 µm) so that with relatively short tracks (62±6 µm compared to about 85 µm on the two other nanopatterns) the growth cones came forward over a distance of 33±4 µm which is about the same distance as reached on the 86 nm patterns or on ALCAM-coated coverslips. To gain deeper insight into the underlying growth cone performance, three different types of growth cone behavior were evaluated: phases of advance, retraction, and pausing (i.e. no substantial move forward or backward) (**Fig. 5e**, Supplemental Information **Fig. S3**). The evaluation of the different phases showed that pauses on 70 nm ALCAM patterns were longer (plus 76% and 51%) and retractions fewer (minus 43% and 55%) than on 54 and 86 nm patterns, respectively, whereas advance phases did not significantly differ on the three nanopatterns. By the reduced number of retractions together with the reduced lateral deviations on 70 nm patterns (see above), however, the growth cones were fully able to counterbalance the slow growth rate on this pattern.

**Figure 6:**
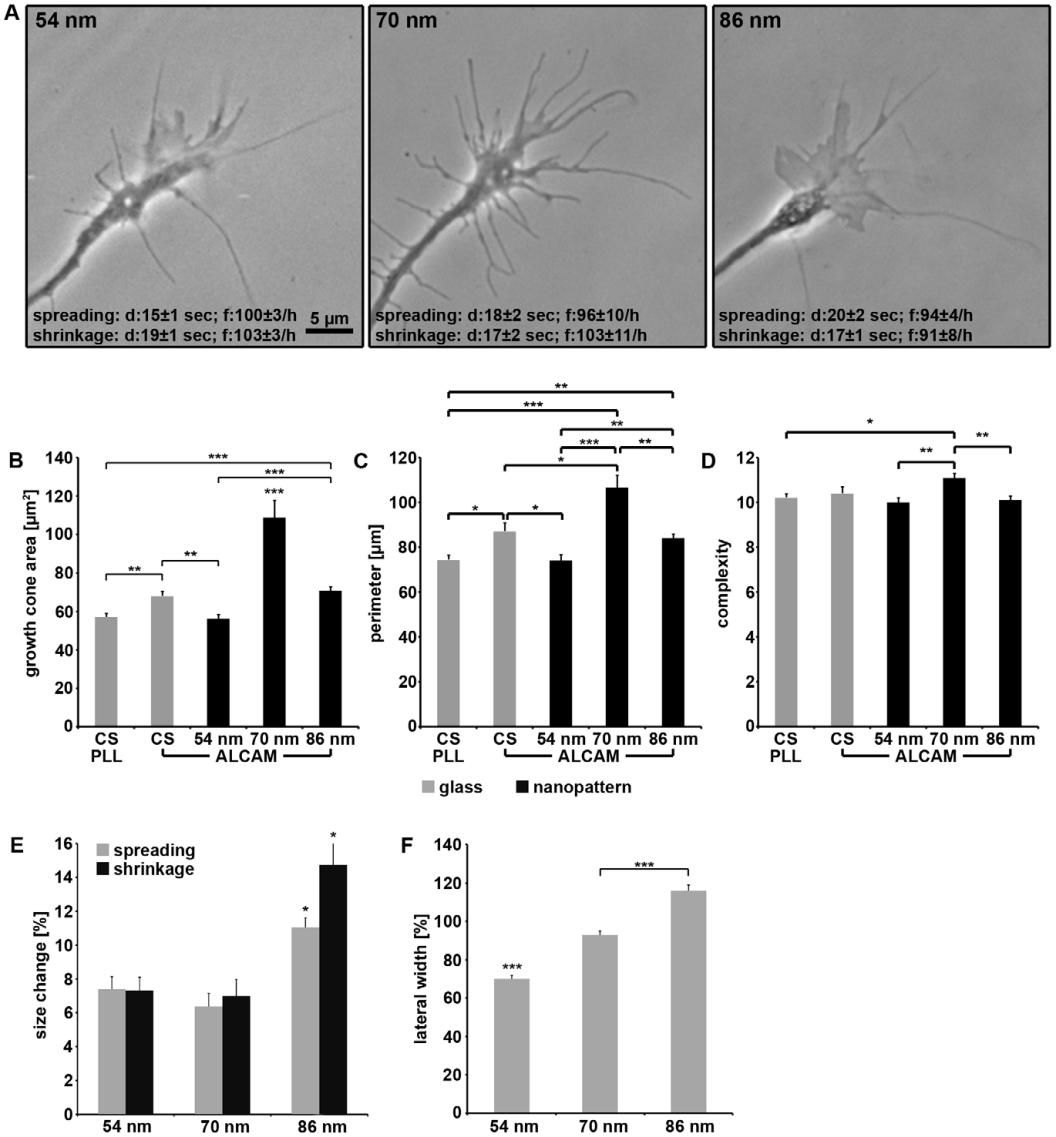
Growth cone dynamics on various substrates. (**A**) Phase contrast micrographs of growth cones on various ALCAM nanopatterns. Note that duration (d) and frequency (f) of growth cone size changes (spreading, shrinkage) do not differ significantly (between p<0.07 and p<0.6) on the various nanopatterns (ten growth cones each). (**B**) Quantification of growth cone area, (**C**) perimeter, and (**D**) complexity (perimeter/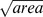) on PLL-/ALCAM-coated glass or on various ALCAM nanopatterns (155 growth cones quantified for each substrate). (**E**) Quantification of change in growth cone size (spreading/shrinkage) expressed as percentage of growth cone area on various ALCAM nanopatterns (ten growth cones each; monitored for 300 sec, every 10 sec). (**F**) Quantification of growth cone width (expressed as percentage of growth cone length) on various ALCAM nanopatterns. Error bars represent SEM; for statistical analyses, two-way ANOVA followed by post-hoc comparisons using two-tailed Student's t test with Bonferroni-Holm corrections were performed (***P<0.001, **P<0.01, *P<0.05).

**Figure 7:**
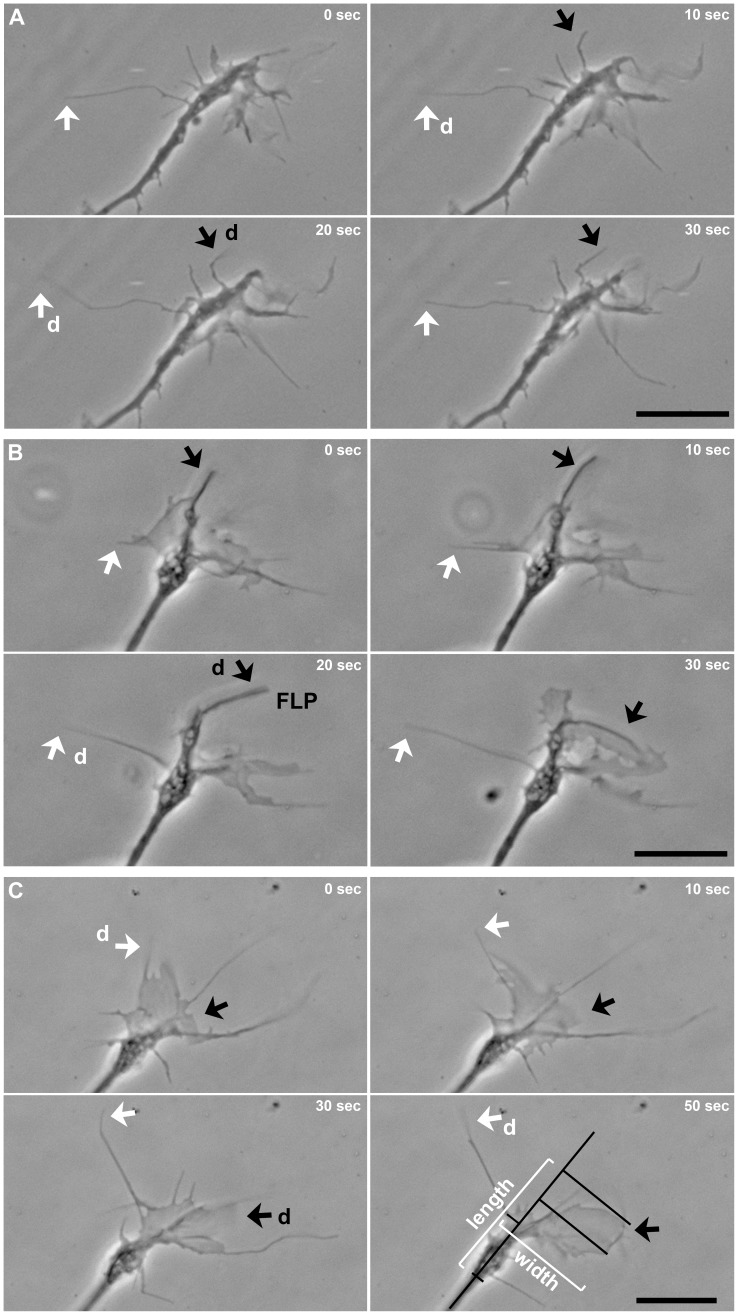
Dynamics of growth cone protrusions on nanopatterns. (**A**) Time-lapse phase contrast micrographs of a growth cone on an 86 nm ALCAM pattern show two filopodia (white and black arrows) which detach (d, move out of focus; 20 sec) from the substratum and reattach (again in focus; 30 sec). (**B**) Another example of a filopodium (white arrow) and a stronger, finger-like protrusion (FLP, black arrow) detaching and reattaching; (**C**) also a lamellipodium (black arrow) de- and reattaches. Length and width of a growth cone were determined as indicated by white and black lines. Scale bars, 10 µm.

The analysis of growth cone morphology and behavior (**Fig. 6**) revealed that growth cones were larger (plus 95% and 54%) on 70 nm ALCAM patterns compared to those on 54 and 86 nm patterns, respectively (**Fig. 6a, b**). The growth cones on 70 nm patterns in addition possessed numerous protrusions, resulting in a larger perimeter compared to those on 54 and 86 nm patterns (plus 45% and 26%, respectively)(**Fig. 6c**). Together this causes an increase in growth cone complexity on 70 nm patterns (plus 12% and 10%), a hallmark of an enhanced screening activity (**Fig. 6d**). This finding points to the growth cone sensing the meager support of axon elongation and branching by the 70 nm pattern and increasing exploration to escape this environment. Analysis of growth cone shrinkage and spreading (i.e. a size change of more than 5% in 10 sec) showed that both types of events lasted about 15–20 sec and each happened almost 100 times per hour on all three nanopatterns (**Fig. 6a**). On 54 and 70 nm ALCAM patterns, the changes in growth cone area caused by spreading or shrinkage were about 7% in 10 sec. In contrast, on 86 nm patterns, size changes are more pronounced (11±0.5% and 15±1.5% in 10 sec for spreading and shrinkage, respectively) (**Fig. 6e**, Supplemental Information **Fig. S4**). This is in accordance with the observation that growth cones on 86 nm patterns changed direction (8.1±2/h) - a process which requires partial spreading and shrinkage - more often than on the other nanopatterns (54 nm: 6.3±1.4/h; 70 nm: 3.8±1.5/h). Together, the data reveal the dependence of growth cone stability on the density of attachment sites offered by the substrate as also observed for detachment events (see below, **Fig. 7**).

To investigate the lateral exploration behavior of growth cones, their width was determined in correlation to their length (every 10 sec for 5 min) (**Fig. 6f**, see also **Fig. 7**). Growth cones showed a moderate lateral extent on 54 nm ALCAM patterns (59±4% longer than wide), more width on 70 nm patterns (14±2%), and were as wide as long on 86 nm patterns. These findings indicate a classical dose-response dependency of growth cone slimness on the density of attachment sites (**Fig. 6f**). The dynamics of the formation/retraction of lateral growth cone protrusions were equal on all three nanopatterns (frequency: 1.0–1.2/min, duration: 13–17 sec, change in width: 7–10 µm). The extent of the lateral gain or loss thus is independent of the average growth cone width displayed on the various patterns (54 nm: 12±0.4 µm, 70 nm: 20±0.8 µm, and 86 nm: 17±0.6 µm). Together, the data indicate that - although growth cone morphology (shape and size) depends on the density of attachment sites offered - lateral exploration events (occurrence and extent) are rather regulated by an intrinsic program than being a reaction to substrate properties.

Time-lapse analyses also revealed that a local loss of substrate attachment of growth cones was possible without retraction of the detached part: Filopodia as well as lamellipodia were observed to detach rapidly and to move upwards, away from the substrate (**Fig. 7**). This phenomenon was found for growth cones on conventional substrates (on PLL: 42±6/h) as well as on ALCAM nanopatterns (see below). Detached filopodia were observed to float in the medium up to 250 sec and settled down on the substrate again without shrinkage, pointing to internal structures providing the necessary stiffness to these filopodia. De- and reattachment was also observed for lamellipodia; this was always accompanied, however, by changes in shape, pointing to a re-arrangement of the cytoskeletal system which is known to be more complex in lamellipodia (meshwork) than in filopodia (bundles). The use of nanopatterns revealed that the frequency of such detachment events (27±6/h on 54 nm, 44±5/h on 70 nm, and 56±6/h on 86 nm patterns) negatively correlates with the density of attachment sites in a dose-response fashion.

### Impact of ALCAM density on non-neuronal DRG cells

**Figure 8:**
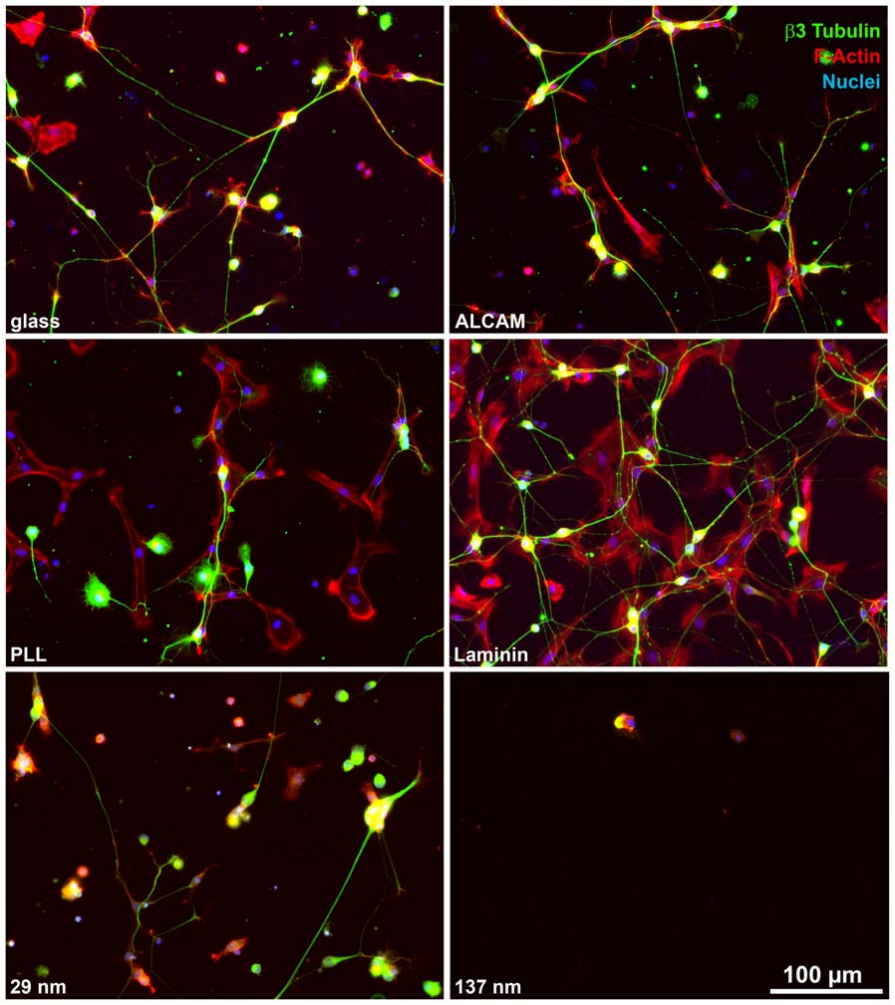
Adhesion of DRG cells to conventional substrates and nanopatterns. Immunofluorescence labeling shows neurons (identified by β3 tubulin staining) and non-neurons (visualized by F-actin labeling) attached to uncoated glass or glass coated with various molecules and to 29 nm or 137 nm nanopatterned ALCAM substrates.

The role of the substrate ALCAM density for the attachment of non-neuronal DRG cells (immature glial cells and mesenchymal cells), which make up about half of the cells in DRG single cell cultures, was investigated since this is of high relevance for the design of regeneration paradigms. DRG single cell cultures were triple-labeled after 1 div with a nuclear marker (DAPI), a neuron-specific marker (β3-tubulin), and an F-actin marker (phalloidin) which visualizes all DRG cells (neurons and non-neurons) (**Fig. 8**). Quantitative evaluation (Supplemental Information **Fig. S5**) revealed that DRG cells (non-neurons and neurons) adhered to ALCAM (set to 100±3%) to a lesser degree than to laminin (162±7%; p≤1*10^−12^) or to PLL (121±5%; p≤0.05). Within the DRG cell population, the fraction of non-neuronal cells which attached to ALCAM substrate (54±16%) was lower than on PLL- (66±16%; p≤2*10^−10^) or on laminin-coated coverslips (62±9%; p≤0.03). This is due to a reduced number of non-neuronal cells adhering on ALCAM-coated coverslips (114±4/mm^2^) than on PLL- (175±9/mm^2^) or laminin-coated coverslips (212±10/mm^2^) i.e. not to an increase in attachment of neurons to ALCAM coverslips. Coverslips coated with five different concentrations of ALCAM (1–50 µg/ml) showed that the attachment of non-neuronal DRG cells (1 µg/ml: 95±9/mm^2^; 50 µg/ml: 114±4/mm^2^) did not correlate with the concentration of substrate ALCAM. This is in contrast to the adhesion of DRG neurons (1 µg/ml: 55±4/mm^2^; 50 µg/ml: 98±3/mm^2^; p≤2*10^−10^) which clearly depended on the substrate ALCAM concentration [9]. This points to a masking of the correlation of the adhesion of non-neuronal cells to ALCAM by shed proteins, as also indicated by the complete absence of any cell adherence to PEG-coated coverslips (see Discussion). The use of ALCAM nanopatterns showed that the attachment of non-neuronal cells clearly correlated with the density of ALCAM molecules, increasing to about double numbers of attached cells from 86 nm (38±2/mm^2^) to 29 nm spacing (77±8/mm^2^, p≤2*10^−10^) (Supplemental Information **Fig. S5**); on 137 nm patterns, hardly any cells adhered at all (7±1/mm^2^). Together, these findings show that non-neuronal cells adhere less to ALCAM than to other substrates, and attach to ALCAM-nanopatterns in a density-dependent manner.

## Discussion

The aim of this study was to investigate the responses of neurons and non-neurons to substrate ALCAM which is of importance for the use of this CAM in regeneration-enhancing clinical paradigms (see below). ALCAM was offered in a highly defined spacing/orientation and without disturbance by unknown proteins. Previous studies using DRG and retinal ganglion cells cultured on glass coverslips coated with ALCAM [11], [17], [19] indicated a potential axon growth promoting capacity of this CAM. Such experiments employing conventional coating are impaired, however, by proteins shed by the cultured cells and deposited on the glass surface. Binding of considerable amounts of released proteins, including such mediating cell adhesion and axon growth, to glass coverslips has been repeatedly shown [26], [27], [28], [29], [30] and render a stringent interpretation of the collected data basically impossible. In this study, cell attachment and axon growth on uncoated glass (i.e. glass which was not coated by the experimenter) is observed which clearly indicates the presence of deposited proteins as revealed by PEG-coated coverslips where deposition is effectively prevented and no cells adhere [6], [31]. The effect of the protein deposition on glass coverslips is so dominant that it masks the density-dependency of the cellular responses which only become visible on nanopatterns (which are all PEG-passivated). Using passivated nanopatterns, we could previously show the adhesion of neurons to ALCAM [9]. We here show for the first time that ALCAM per se - without any support by other proteins - is also able to mediate adhesion of non-neuronal cells as well as to regulate axon elongation/branching and govern growth cone dynamics.

Another important property of the nanopatterned substrates is the physiological orientation of the bound protein (domain) which is alternatively only achieved by (transfected) cells presenting the protein of interest. An advantage of the CAM presentation in the plasma membrane is a physiological membrane mobility [32]. Highly problematic, however, is the impact of other – known and unknown - plasma membrane proteins which assist or impede (by cis-interactions) the cellular effect elicited by the CAM of interest. Alternatively these plasma membrane proteins might exert a positive or negative effect on the cellular responses by themselves, which could be modulated by the CAM of interest (e.g. via intracellular signaling switches). The nanopattern experiments show that substrate-bound ALCAM trans-interacts with DRG cells, with the expression analysis strongly suggesting that such interactions also take place in vivo between fasciculating DRG axons (in the central/peripheral nerves) and somata (in the DRGs). Most likely, these adhesive contacts between neurons are based on homophilic interactions, since ALCAM is known to bind to ALCAM [12]. Non-neuronal DRG cells (future Schwann and satellite cells and DRG-ensheating mesenchymal cells) do not display detectable levels of ALCAM (not shown). Hence, heterophilic trans-interactions between substrate ALCAM and until now largely unknown proteins in the plasma membrane of these cells have to take place; so far, only two heterophilic interaction partners of ALCAM (L1 and CD6) have been identified [33]. The heterophilic interactions of non-neuronal DRG cells are considerably weaker than the homophilic ones, as these cells attach to a lesser degree to ALCAM substrates than to conventional substrates such as laminin.

The highly defined density of the presented protein - which can be varied - is the most obvious advantageous feature of nanopatterns [6], [7]. This cannot be achieved by coating coverslips with concentration series of protein solutions due to the hardly controllable protein deposition (amount and homogeneity). Only the use of nanopatterns revealed in this study that growth cone responses show classical density (“dose”) dependence. With increasing ALCAM density, the growth cone acquires a slimmer shape, i.e. reduces its left and right screening range, while low ALCAM densities lead to an increased lateral exploration extent. This could contribute to effective fascicle formation in vivo as the highest densities of ALCAM are present in the DRG central/peripheral nerves. DRG axons which grow on other DRG axons would thus benefit from a reduction in lateral probing, keeping them in contact with the other axons and preventing straying away. Growth cones in vivo, which track on axon bundles, indeed have a slim morphology and restricted lateral probing activity [34]. Not only growth cone shape but also dynamics depends on ALCAM density: With decreasing substrate ALCAM density, growth cone size changes caused by shrinkage (and subsequent spreading) are more pronounced and detachments of protrusions are more frequent. Moreover, drastic shrinkage events (more than 30% area loss within 10 sec) were observed only on 86 nm patterns (19 such shrinkages/h). Such growth cone reactions indicate a weak substrate attachment, which is most likely directly due to the lower ALCAM density on 86 nm, providing only one-third of the number of adhesion points compared to 54 nm patterns. Conceivably, also a weakening of the cytoskeleton of the cells on the low ALCAM density substrates could underlie the growth cone shrinkage/detachment. This appears, however, highly unlikely since it would not allow for the observed fast growth cone spreading and the long-term stiffness of the detached protrusions.

In contrast to the clear dependence of growth cone behavior on the density of substrate ALCAM, axonal reactions did not respond in a dose-response fashion. The number of axons formed by a DRG neuron was ALCAM independent, indicating a genetically determined program which is triggered above a minimum ALCAM density. This notion is also supported by our observation that 1–2 axons were formed per neuron in vitro on all substrates tested, which is close to the number of axons formed by a DRG neuron in vivo (two). Also the advance of the axons did not depend on ALCAM density (distance covered and duration/frequency of advance phase). Interestingly, it is due to counterbalancing that both features did not differ on the various ALCAM nanopatterns: the track length was increased depending on extent of the lateral deviations and thus covers the same distance; retractions and pauses compensate each other thereby keeping advance phases equal on all nanopatterns. Some responses were lowest on the 70 nm patterns and not on the least dense ones: axon growth (which is in accordance with a previous study, where neuritogenesis and total neurite length were poorest on 70 nm [9]), axon branching, and growth cone velocity. To substantiate this observation, more nanopatterns with intermediate spacing (between 54 nm and 70 nm as well as between 70 nm and 86nm) have to be analyzed. On the basis of the data obtained so far, it can only be speculated about the underlying molecular mechanisms, which might involve the cortical cytoskeleton anchorage of ALCAM [9].

Offered as a nanopattern, ALCAM is able to support axon elongation even at densities far below physiological levels (i.e. the density as presented in the axonal plasma membranes) while attachment of non-neuronal cells is significantly decreased on these patterns. This is due to the regular spacing and the correct amino-carboxyl terminal orientation of the CAM as well as the absence of other (deposited) proteins. Low-density ALCAM nanopatterns might thus become a useful tool for the enhancement of axonal regeneration by an implant bridging the gap between the proximal and distal stumps of injured nerves. Attachment of non-neuronal cells is a severe problem of conventional implants which are coated with proteins rather promiscuously mediating cell attachment (laminin/ECM, e.g.). The vast attachment of non-neuronal cells to such proteins makes these inaccessible for regenerating axons which thus do not extend on the implant. Since the sparse 86 nm pattern promotes axonal growth to the full extent (i.e. to the same degree as the 29 nm pattern which is almost tenfold denser) and at the same time only mediates a reduced attachment of non-neuronal cells (half the cell number compared to 29 nm patterns) the 86 nm pattern turned out to be the most suitable of the substrates tested in this study for the use as an implant. Further steps improving the properties of this nanopattern, e.g. offering an optimized (domain-tailored) form of ALCAM, will optimize it for a future application in clinical paradigms.

## Materials and Methods

### Animals, antibodies, and reagents

Fertilized white leghorn chicken eggs were obtained from a local provider (LSL, Dieburg, Germany). Chick embryos were handled in accordance with national guidelines; according to the german animal protection act embryos are not considered as animals before hatching (§7). Embryos were incubated at 37°C in a humidified atmosphere and sacrificed by decapitation as approved by the animal protection officer of the University of Heidelberg. Antibodies used were rabbit polyclonal antibodies against ALCAM [19], mouse monoclonal antibody against NgCAM (1E12 [35]), mouse monoclonal antibody against β3-tubulin (Tuj1, Covance), Alexa488-conjugated goat-anti-rabbit and Alexa546-conjugated goat-anti-mouse polyclonal antibodies (Invitrogen). Texas-red-conjugated phalloidin, laminin, poly-L-lysine (PLL), 4′,6-Diamidino-2-phenylindol (DAPI), and Trypsin were purchased from Sigma. The recombinant, histidine-tagged extracellular domain of ALCAM was produced as described [10].

### Immunofluorescence procedures

Immunofluorescence labeling was performed as described [18]. In brief, sections of chick embryos of different ages were fixed by 4% paraformaldehyde and 11% sucrose in PBS for 24 h, and in 25% sucrose in PBS for additional 12 h (E5, E7 embryos), 24 h (E9, E11 embryos) or 48 h (E13–E20 embryos). The specimens were embedded (Tissue Tec O.C.T., Sakura, Netherlands), sectioned with a cryostat (12 µm, Reichert and Jung, Germany), and immuno-labeled as described [36]. Micrographs were taken by an inverted fluorescence microscope (Axiovert 200M, Zeiss) equipped with a digital camera (AxioCam Rev3, Axiovision software, Zeiss) and annotated with Adobe Photoshop CS2. For quantification of ALCAM levels, the region of interest was outlined in ImageJ (NIH) and the immunofluorescence brightness (grey values) determined. Cell culture specimens were fixed by 4% paraformaldehyde/PBS for 60 min, permeabilized for 10 min with 0.1% Triton-X100 in PBS, and stained as described [37].

### Coating of glass surfaces and preparation of nanopatterns

Cleaning and coating of glass coverslips was performed as described [18]. In brief, coverslips (Merck) were cleaned with hydrochloric acid/ethanol and coated either with PLL (40 µg/ml), PLL/laminin (50 µg/ml), PLL/ALCAM (50 µg/ml) or not coated at all; for concentration series, 1–50 µg/ml ALCAM in PBS were used. Hexagonally arranged gold nanodots (distances 29–137 nm) were fabricated by a technique based on the self-assembly of gold-loaded diblock copolymer micelles [6], [7], [38]. A sample of each batch of fabricated nanopatterns was checked for their quality by electron microscopy. The 70 nm patterns were produced using exactly the same technique as the other patterns. We saw the 70 nm phenomenon on all batches (more than ten) we obtained. The space between the gold nanodots was passivated by poly-ethylene glycol (PEG) for prevention of protein deposition to the glass surface. To link ALCAM to the gold nanodots, nanosubstrates were equipped with a nickel/nitrilotriacetic (NTA)-thiol linker as described [39]. The specimens were then incubated overnight at 4°C with the recombinant histidine-tagged extracellular domain of ALCAM (1 µg/ml in PBS), followed by thorough washing (5×15 min in PBS).

### Cell culture

Dorsal root ganglia (DRG) single cell cultures were prepared as described [40]. In brief, embryonic day (E) 9 chick DRGs were isolated, incubated for 10 min in Hank's buffer containing 1 mg/ml Trypsin at 37°C, mechanically dissociated by trituration through a Pasteur pipette, and resuspended in DMEM/F12 (Sigma) supplemented with 1% N2 (Invitrogen), 10 ng/ml nerve growth factor (Invitrogen), and 0.1% Gentamycin (Invitrogen). To reduce number of non-neurons, the cell suspension was pre-incubated for 90 min in an uncoated tissue culture dish to make these cells attach [41]. The non-attached cells were collected, seeded (2×10^4^ cells in 200 µl), and cultured on coverslips or nanopatterns for 24 h; the sparse culture allowed for the extension of axons without contact to other neurons.

### Quantification of cell attachment and axon properties

Numbers of cells attached to the various substrates were determined by evaluation of random optic field micrographs (size of optic field: 0.14 mm^2^, 20 per coverslip or nanopattern). The total number of attached cells was determined by counting DAPI-stained nuclei; neurons were identified by β3-tubulin labeling. Axon number, length, and branching of (randomly photographed) neurons were measured using ImageJ; only axons longer 30 µm were included in these measurements. For quantification of the growth cone morphology, the growth cone neck was determined (the site where the double diameter of the axon is reached). Area and perimeter of the growth cone was quantified without filopodia (core growth cone) to exclude the substantial interference of these very dynamic structures with the perimeter determination. As a measure for growth cone complexity, the ratio of perimeter and square root of area was calculated. Growth cone length was determined by fitting a line to the distal-most axon section (10 µm behind the growth cone neck) and measuring the maximal extension of the core growth cone reached along the forward-projection of this line (see **Fig. 7c**). The width of the core growth cone was determined by measuring the maximal extension perpendicular to the left and right of this projection line and growth cone slimness by the length/width ratio. Statistical analyses were performed using two-way ANOVA followed by post-hoc comparisons using two-tailed Student's t test with Bonferroni-Holm corrections.

### Time-lapse microscopy, axon tracking, and growth cone dynamics

Glass coverslips or nanopatterns were fitted into a hole drilled into Petri dishes [42]. Time-lapse studies were performed using an inverted microscope (Axiovert 200M, 63× objective, Zeiss) equipped with a digital camera (AxioCam, Zeiss) and a self-made incubation chamber with thermo- and CO_2_ regulation. Phase-contrast micrographs were taken at 10 s intervals for 1 h. Growth cone advance was tracked (using ImageJ, NIH) by locating the position of the growth cone neck (see above) every minute for 1 h (i.e. 600 positions evaluated per substrate). The coordinates were transferred to Microsoft Excel 2007 and the growth cone positions analyzed. To quantify growth cone velocity and advance, the overall growth direction, i.e. the line of best fit, was calculated (LINEST function, considering all growth cone positions during the entire observation period). Advance was defined as growth cone movement within 1 min relative to the line of best fit of ≥1 µm, retraction as ≤−1 µm, and pause as <1 µm to >−1. For the determination of growth cone velocity, retractions were not included in the evaluation. The dynamics of growth cone size were analyzed by determining of the growth cone area every 10 sec over a period of 5 min. Statistical analyses were performed using two-way ANOVA followed by post-hoc comparisons using two-tailed Student's t test with Bonferroni-Holm corrections.

## Supporting Information

Figure S1 (**A**) **Schematic of ALCAM molecules in plasma membrane and on nano-patterned substrate.** The extracellular domain of ALCAM (ec-ALCAM, length: 20 nm, diameter: 4.5 nm) is coupled to the gold dot (diameter 5 nm, yellow) in physiological orientation, i.e. the amino-terminus (N) is directed toward the opposing cell membrane. The polyethylene glycol (PEG) layer between the gold dots leaves the monothiol-NTA linker (red square) accessible and prevents deposition of proteins. The substrate ec-ALCAM trans-interacts with ALCAM molecules in the plasma membrane (p.m.) which contain in their carboxyl terminal (C) domain a potential binding site for cytoskeletal linker proteins (ERM). c.c. = cortical cytoskeleton, c.p. = cytoplasm (**B**) Immunofluorescence staining of ALCAM on nano-patterned substrates selectively labels the area containing the ec-ALCAM presenting gold dots and visualizes the straight border (dip line) to the area containing no gold dots. (**C**) DRG cells cultured for 24 h on ALCAM nanopatterned substrates and immunofluorescence labeled for ALCAM, exclusively attach to the ec-ALCAM presenting gold dot-containing area. Note that also axon extension is restricted to this area with axons avoiding the ec-ALCAM free area by turning away and/or growing parallel to the border (arrow heads). (**D**) Scanning electron microscopy revealed the regular gold dot distribution on the various nanopatterns.
(JPG)

Figure S2 **Three growth cone tracks on each ALCAM nanopattern (additional tracks to** **Fig. 5** **) as observed by time-lapse phase contrast microscopy.** Each dot represents the position of the growth cone neck, localized every minute for one hour.
(JPG)

Figure S3 **Growth cone behavior, i.e. advance, pause, and retraction (green: >1 µm/min, yellow: −1 to +1 µm/min, and red: <−1 µm/min, respectively) of ten different axons on PLL- or ALCAM-coated glass coverslips plotted for 60 min.** Velocity, duration, and frequency of the three types of behavior were determined with respect to the line of best fit.
(JPG)

Figure S4 **Growth cone dynamics of five different growth cones on various ALCAM nanopatterns monitored for 5 min.** The degree of spreading (plus values) and shrinkage (minus values) of the growth cones is plotted (as a percentage of growth cone area) every 10 sec. Drastic shrinkage events (more than 30% area loss within 10 sec) were almost only observed on 86 nm ALCAM patterns.
(JPG)

Figure S5 **(A, B) Quantification of attachment of (A) DRG cells or (B) non-neuronal cells to glass coated with PLL, ALCAM, or laminin.** (**C, D**) Quantification of attachment of (C) DRG cells or (D) non-neuronal cells to uncoated glass (UCG) or glass coated with PEG or increasing ALCAM concentrations. (**E, F**) Quantification of attachment of (E) DRG cells or (F) non-neuronal cells to various ALCAM nanopatterns. Error bars represent SEM; ***P<0.001, **P<0.01, *P<0.05.
(JPG)

Movie S1 **Time-lapse movie of a growth cone on a 54 nm pattern functionalized with ALCAM.** Images were taken after 1 day in culture every 10 sec. The movie playback is at 5 frames/sec (i.e. 50× speed).
(AVI)

Movie S2 **Time-lapse movie of a growth cone on a 70 nm pattern functionalized with ALCAM.** Images were taken after 1 day in culture every 10 sec. The movie playback is at 5 frames/sec (i.e. 50× speed).
(AVI)

Movie S3 **Time-lapse movie of a growth cone on a 86 nm pattern functionalized with ALCAM.** Images were taken after 1 day in culture every 10 sec. The movie playback is at 5 frames/sec (i.e. 50× speed).
(AVI)

Movie S4 **Time-lapse movie of a growth cone on a coverslip coated with PLL/ALCAM.** Images were taken after 1 day in culture every 10 sec. The movie playback is at 5 frames/sec (i.e. 50× speed).
(AVI)

Movie S5 **Time-lapse movie of a growth cone on a coverslip coated with PLL.** Images were taken after 1 day in culture every 10 sec. The movie playback is at 5 frames/sec (i.e. 50× speed).
(AVI)
