# Supplementary figures and images for: Depending on Its Nano-Spacing, ALCAM Promotes Cell Attachment and Axon Growth
Source: PLoS One. 2012 Dec 10;7(12):e40493. doi: 10.1371/journal.pone.0040493 (PMC3518477; doi:10.1371/journal.pone.0040493)

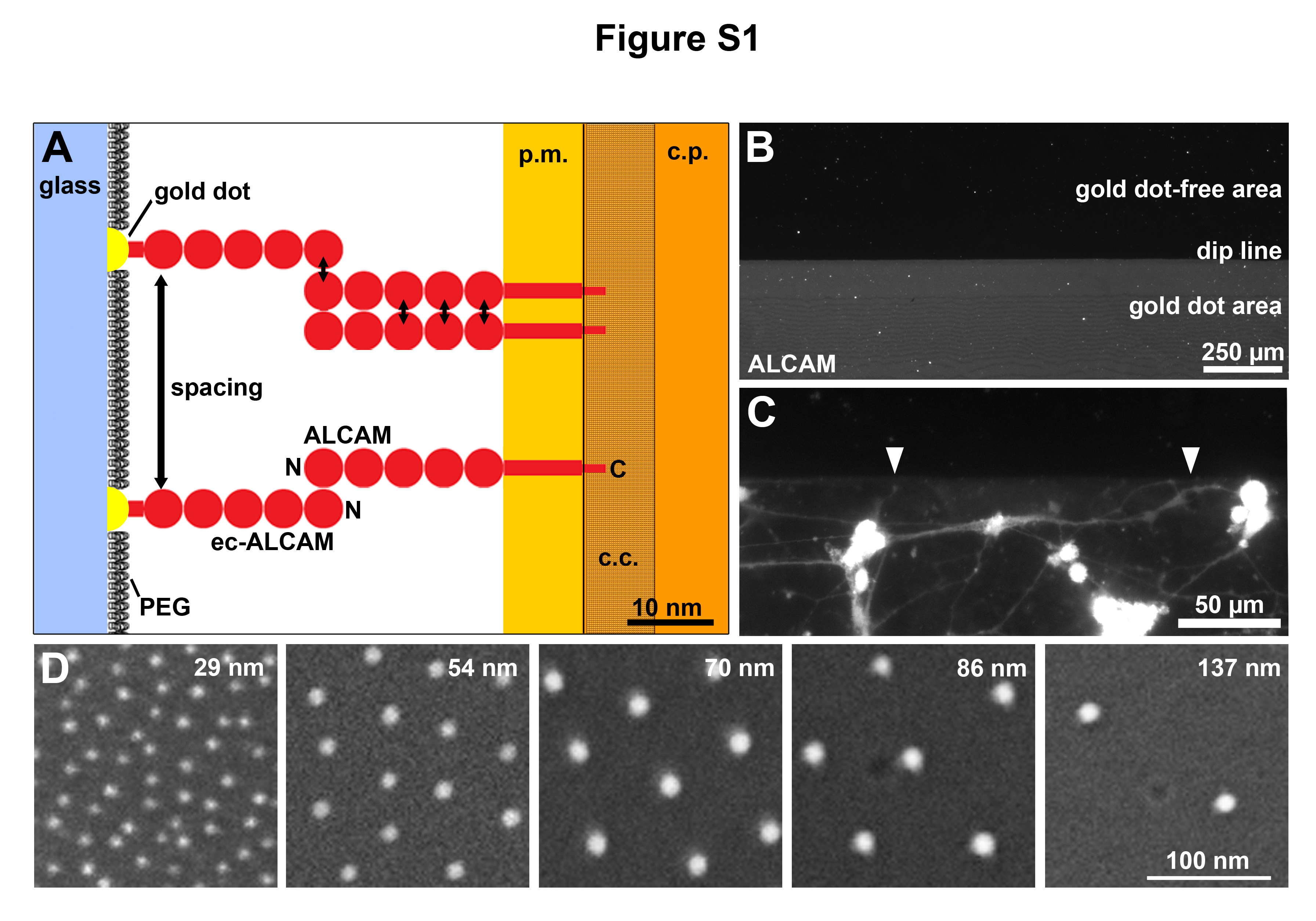

Supplement: Figure S1 — (A) Schematic of ALCAM molecules in plasma membrane and on nano-patterned substrate. The extracellular domain of ALCAM (ec-ALCAM, length: 20 nm, diameter: 4.5 nm) is coupled to the gold dot (diameter 5 nm, yellow) in physiological orientation, i.e. the amino-terminus (N) is directed toward the opposing cell membrane. The polyethylene glycol (PEG) layer between the gold dots leaves the monothiol-NTA linker (red square) accessible and prevents deposition of proteins. The substrate ec-ALCAM trans-interacts with ALCAM molecules in the plasma membrane (p.m.) which contain in their carboxyl terminal (C) domain a potential binding site for cytoskeletal linker proteins (ERM). c.c. = cortical cytoskeleton, c.p. = cytoplasm (B) Immunofluorescence staining of ALCAM on nano-patterned substrates selectively labels the area containing the ec-ALCAM presenting gold dots and visualizes the straight border (dip line) to the area containing no gold dots. (C) DRG cells cultured for 24 h on ALCAM nanopatterned substrates and immunofluorescence labeled for ALCAM, exclusively attach to the ec-ALCAM presenting gold dot-containing area. Note that also axon extension is restricted to this area with axons avoiding the ec-ALCAM free area by turning away and/or growing parallel to the border (arrow heads). (D) Scanning electron microscopy revealed the regular gold dot distribution on the various nanopatterns. (JPG) [file pone.0040493.s001.jpg]

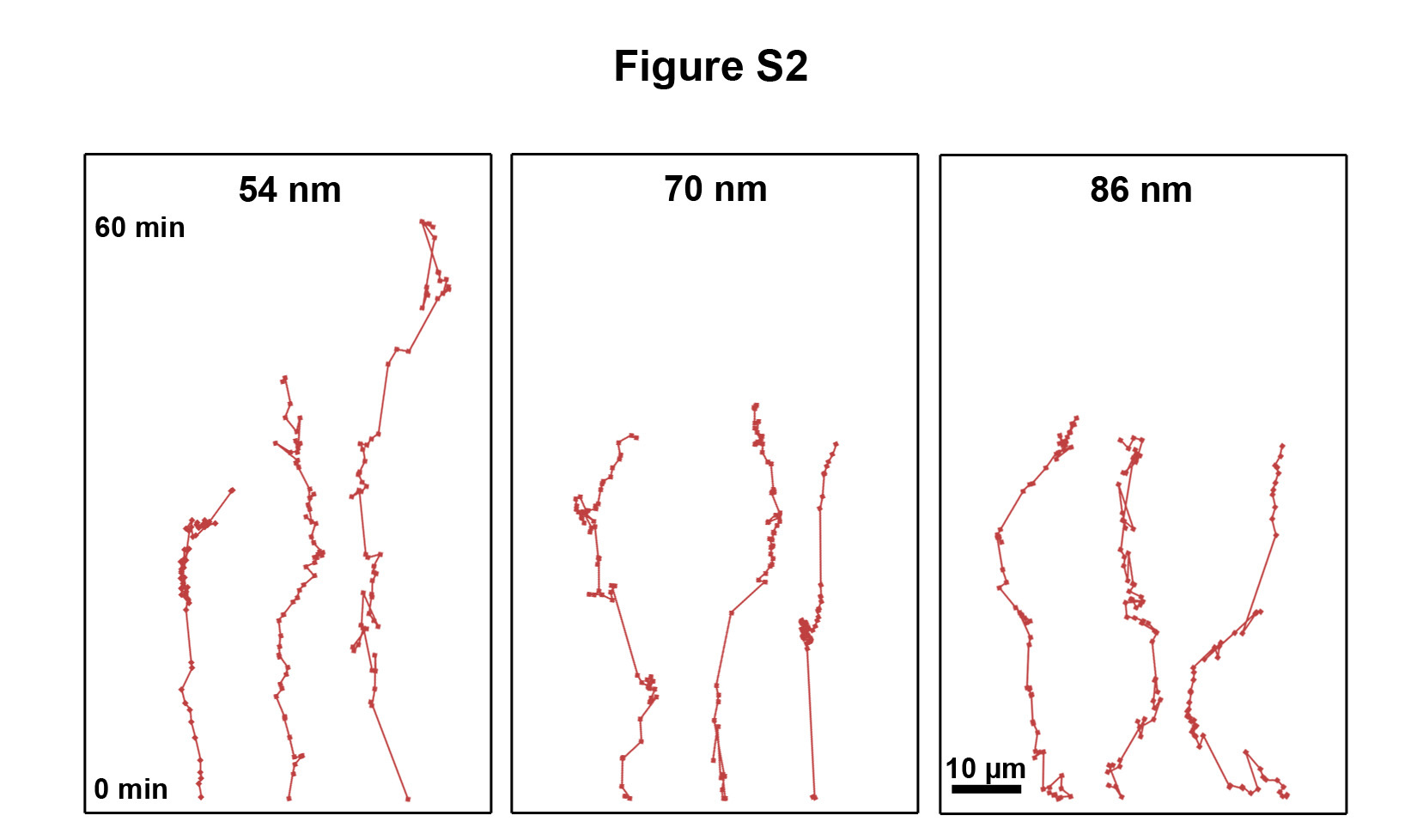

Supplement: Figure S2 — Three growth cone tracks on each ALCAM nanopattern (additional tracks to Fig. 5 ) as observed by time-lapse phase contrast microscopy. Each dot represents the position of the growth cone neck, localized every minute for one hour. (JPG) [file pone.0040493.s002.jpg]

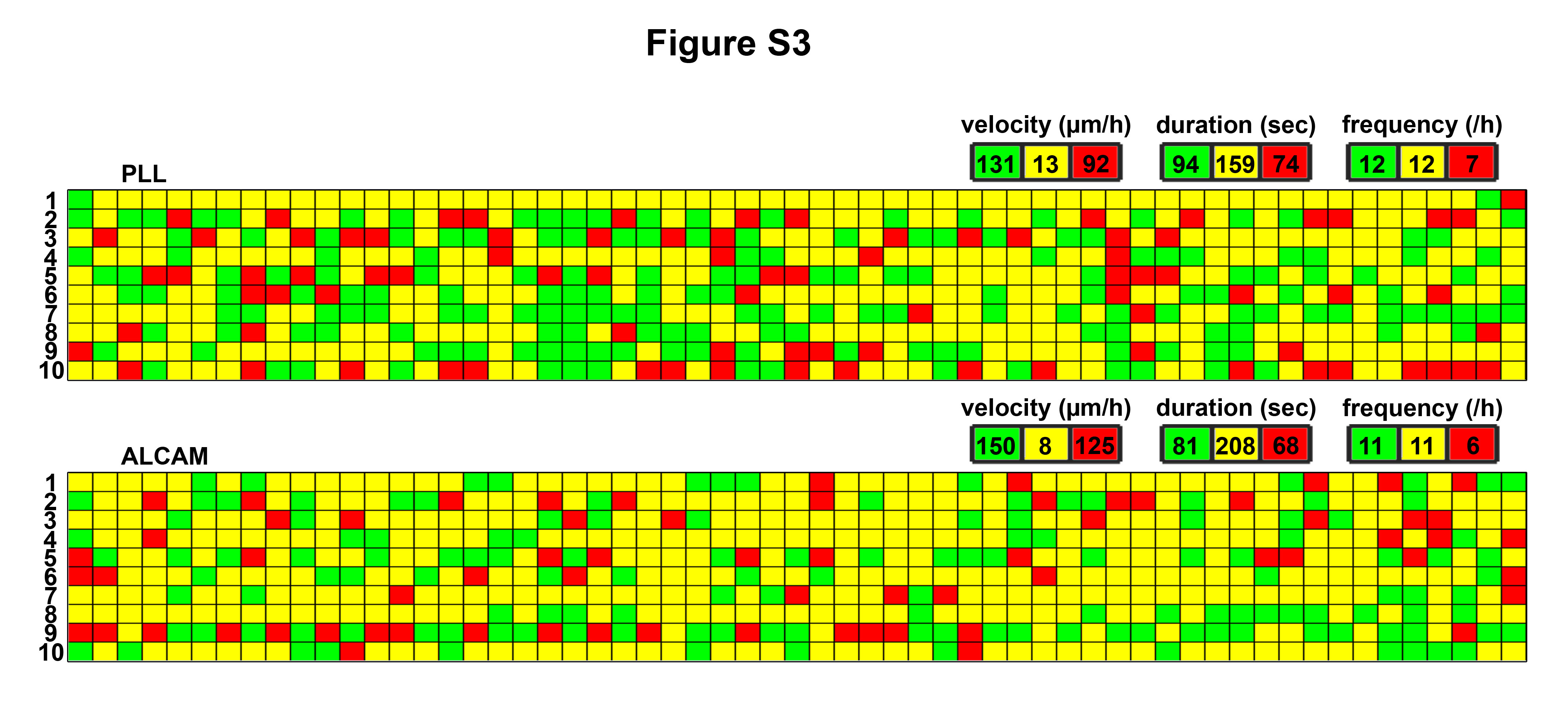

Supplement: Figure S3 — Growth cone behavior, i.e. advance, pause, and retraction (green: >1 µm/min, yellow: −1 to +1 µm/min, and red: <−1 µm/min, respectively) of ten different axons on PLL- or ALCAM-coated glass coverslips plotted for 60 min. Velocity, duration, and frequency of the three types of behavior were determined with respect to the line of best fit. (JPG) [file pone.0040493.s003.jpg]

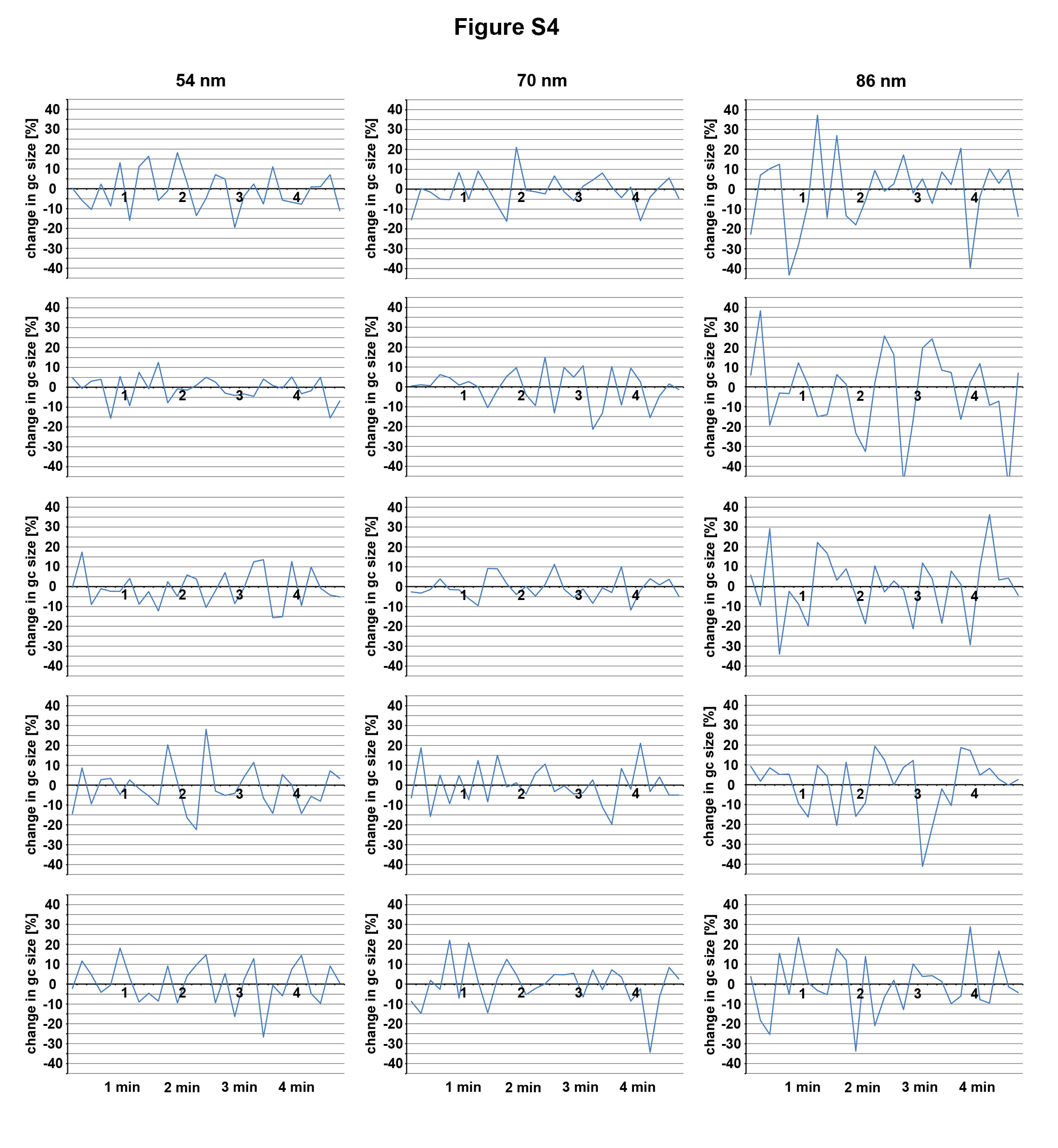

Supplement: Figure S4 — Growth cone dynamics of five different growth cones on various ALCAM nanopatterns monitored for 5 min. The degree of spreading (plus values) and shrinkage (minus values) of the growth cones is plotted (as a percentage of growth cone area) every 10 sec. Drastic shrinkage events (more than 30% area loss within 10 sec) were almost only observed on 86 nm ALCAM patterns. (JPG) [file pone.0040493.s004.jpg]

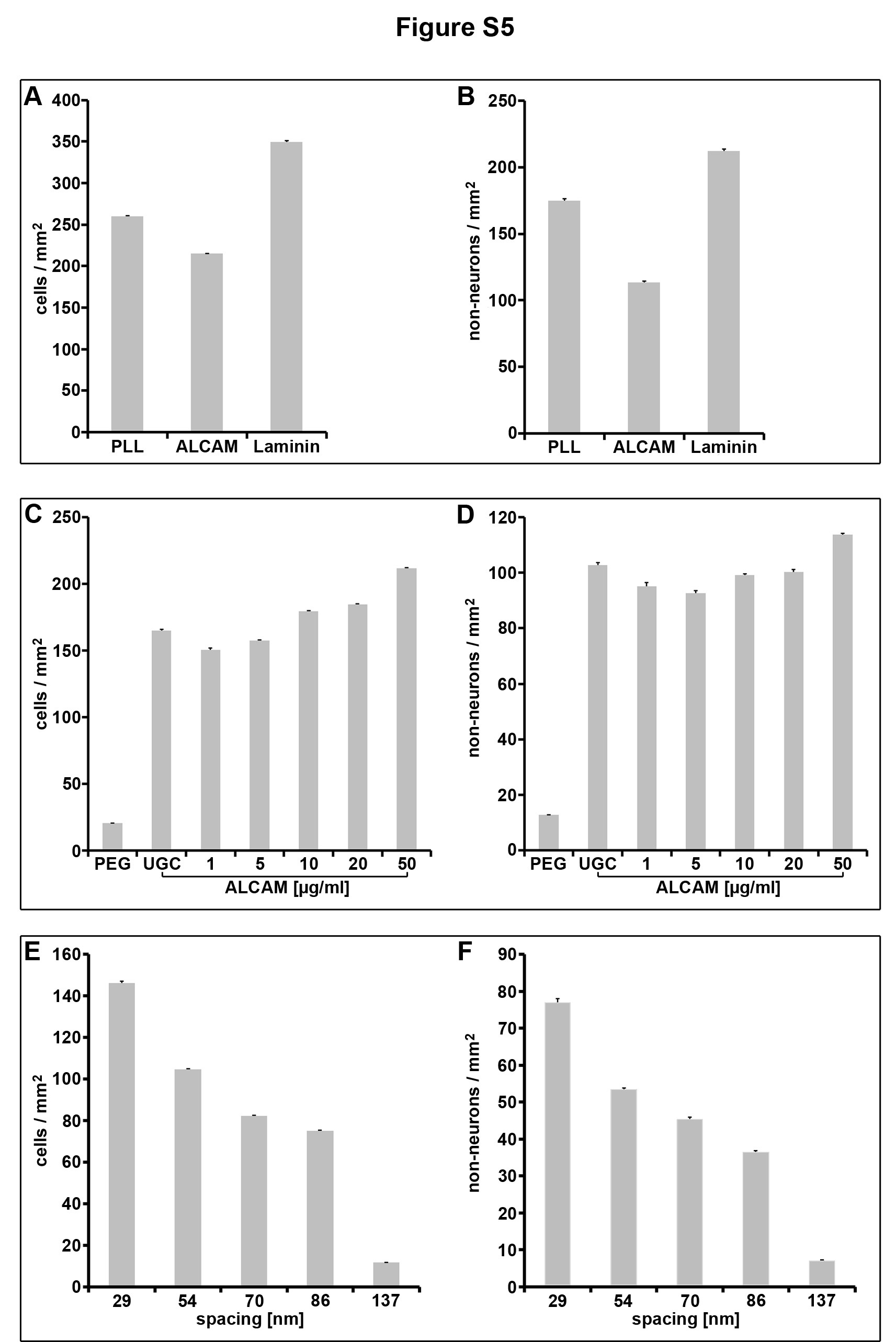

Supplement: Figure S5 — (A, B) Quantification of attachment of (A) DRG cells or (B) non-neuronal cells to glass coated with PLL, ALCAM, or laminin. (C, D) Quantification of attachment of (C) DRG cells or (D) non-neuronal cells to uncoated glass (UCG) or glass coated with PEG or increasing ALCAM concentrations. (E, F) Quantification of attachment of (E) DRG cells or (F) non-neuronal cells to various ALCAM nanopatterns. Error bars represent SEM; ***P<0.001, **P<0.01, *P<0.05. (JPG) [file pone.0040493.s005.jpg]
